# Implicit and Explicit Measurement of Work-Related Age Attitudes and Age Stereotypes

**DOI:** 10.3389/fpsyg.2020.579155

**Published:** 2020-10-06

**Authors:** Verena Kleissner, Georg Jahn

**Affiliations:** Department of Psychology, Chemnitz University of Technology, Chemnitz, Germany

**Keywords:** implicit age attitude, workplace ageism, Implicit Association Test, work-related age stereotypes, dimensions of age stereotypes

## Abstract

Age attitudes and age stereotypes in the workplace can lead to discrimination and impaired productivity. Previous studies have predominantly assessed age stereotypes with explicit measures. However, sole explicit measurement is insufficient because of social desirability and potential inaccessibility of stereotypical age evaluations to introspection. We aimed to advance the implicit and explicit assessment of work-related evaluations of age groups and age stereotypes and report data collected in three samples: students (*n* = 50), older adults (*n* = 53), and workers (*n* = 93). Evaluative age attitudes were measured implicitly with an Implicit Association Test. Regardless of group, age, and condition (neutral or semantically biased stimuli), the results confirm a stable, moderate implicitly measurable preference for younger over older workers. Whereas explicit measures of general age preferences showed no clear age preference, differentiated explicit measures of work-related age stereotypes also revealed stable preferences in all three samples: Younger workers were rated higher on performance and adaptability and older workers were rated higher on competence, reliability, and warmth. The explicit-implicit correlations were relatively low. Although explicit work-related age stereotypes are differentiated, the stable implicitly measured age bias raises concern. We suggest to apply implicit and explicit measures in the field of ageism in the workplace.

## Introduction

In the field of work, both the general attitude (i.e., an evaluation in favor or disfavor of an object; Eagly and Chaiken, 1993) toward members of a certain age group and more specific age stereotypes (i.e., cognitive schemata and beliefs about a group based on age; Ng and Feldman, 2012) influence decisions in hiring and career development, allocation of resources, and social behavior at work. A more positive attitude toward younger than older workers may, for instance, bias someone to shortlist preferably younger applicants for a job interview (e.g., Gringart et al., 2005). Age attitudes and stereotypes can be assessed by explicit methods (e.g., “I prefer younger to older workers”), however, individuals may not report a clearly more positive evaluation of a certain age group because it is deemed inappropriate. Alternatively, it may not be reported explicitly because it could be inaccessible to introspection (i.e., “implicit” age attitude, Levy and Banaji, 2002) but it still might subtly manifest in behavior (see De Paula Couto and Wentura, 2017, for a review on implicit ageism). The Implicit Association Test (IAT; Greenwald et al., 1998) can reveal general attitudes and stereotypes even if they are deliberately not reported or inaccessible. Yet, the IAT provides just a relative indicator of attitudes (e.g., “young more positive than old”) or stereotypes (e.g., “family person” vs. “solitary person”; Kornadt et al., 2016). More differentiated stereotypes (e.g., “older workers are less adaptable but more reliable”) can only be assessed by explicit methods. In the present study, we aim to advance the implicit and explicit assessment of work-related evaluations of age groups and age stereotypes, and we examine how the implicit and explicit measures covary. We further examine the influence of perceiving the aging process as malleable on the implicit measure, since perceiving the aging process as malleable was a potential predictor of more positive stereotypes toward older nurses in a recent study (Kleissner and Jahn, 2020).

Measuring attitudes and stereotypes toward age groups in the field of work comprehensively is important for studying how they influence decisions and social behavior, how they vary, and how they can be changed. In the workplace, the rise in negativity of ageist attitudes (Levy, 2017, see North and Fiske, 2012, for a review on ageism) is particularly challenging. First, due to demographic change, the mean age of the workforce is increasing; therefore, more and more older workers and employees will face negative attitudes and negative age stereotypes. Second, such attitudes and stereotypes are associated with negative outcomes for companies and individuals. They can result in workplace discrimination (Jones et al., 2017), which can be expensive for companies (Chao and Willaby, 2007) because of losses in productivity and anti-discrimination laws (e.g., the ADEA – Age Discrimination in Employment Act in the United States). On an individual level, negative attitudes and negative age stereotypes toward older people may be associated with lower quality of life (Palacios et al., 2009), lower functional health (Levy et al., 2002), and lower performance (Lamont et al., 2015). Discriminatory behavior in the workplace can occur in various areas, such as hiring, firing, training, evaluation, and cooperation (e.g., Abrams et al., 2016; Jones et al., 2017).

Because of the detrimental effects of negative age stereotypes in the workplace, it is important for companies to counteract them in an effective age management. Age should be an integral part of diversity trainings that are particularly important for managers. Managers are role models who represent the company’s culture (Desmette and Gaillard, 2008); therefore, it is vital to train them to avoid ageism (Posthuma and Campion, 2009). The present study demonstrated the presence of explicitly and implicitly measurable age stereotypes and age attitudes in the workplace. While the explicit measurement of work-related age stereotypes with a multidimensional work-related stereotype scale revealed both favorable and unfavorable stereotypes for younger and older workers, the implicit measurement revealed a stable moderate preference for younger over older workers even when the stimulus material was in favor of older workers. Therefore, age diversity trainings should specifically address the risk of implicit age stereotypes and age attitudes that can lead to discrimination and impaired productivity. Managers and employees should be aware of the explicit and implicit nature of age stereotypes to reflect them and make bias-free decisions.

## Theoretical Background

Explicit and implicit processes are assumed to operate in parallel and interact with one another (Strack and Deutsch, 2004). Whereas explicit measures aim at capturing beliefs, implicit measures aim at capturing associative structures (Strack and Deutsch, 2004). Therefore, explicit measures typically apply a direct assessment of attitudes (e.g., a survey), whereas implicit measures take a more indirect approach (e.g., the IAT), thereby minimizing the influence of conscious cognitive processes (e.g., social desirability). Nosek and Smyth (2007) found that two-factor attitude models, representing related but distinct constructs, provided a better fit to the data than treating explicit and implicit measures as measuring distinct constructs or the same construct.

### Explicitly Measured Age Stereotypes in the Workplace

Many studies applied questionnaires for investigating the content of age attitudes in the workplace and found that positive stereotypes about older workers characterize them as more accurate, committed to the job and organization, customer-oriented, reliable, socially competent (Van Dalen et al., 2009, 2010), dependable, loyal (Hassell and Perrewe, 1995), responsible, wise (Gringart et al., 2005), and warm (Krings et al., 2011) compared to younger workers. However, there are many negative stereotypes which characterize older workers as set in their ways, less flexible (Van Dalen et al., 2009, 2010), less willing to change (Loretto et al., 2000), and less adaptable (Gringart et al., 2005). Findings regarding stereotypes about older worker’s productivity, competence, and willingness to train are inconsistent. Krings et al. (2011) found that older workers were seen as less competent than younger workers, whereas Gringart et al. (2005) observed the opposite result, which might be due to different samples or measurements. While Krings et al. (2011) had a broad understanding of competence (including, for example, efficiency, and capability) and let competence rate separately for older and younger workers by business students, Gringart et al. (2005) viewed competence in a narrow sense and measured competence contrastively [e.g., How competent are older (55–70) female workers compared to younger workers?] asking decision-makers for ratings. Van Dalen et al. (2009, 2010) detected that older workers were viewed as less productive, whereas older workers were seen as either more productive elsewhere (Loretto et al., 2000; McGregor and Gray, 2002) or equally productive (Gringart et al., 2005) as their younger counterparts. The assessment of productivity may account for these differences. Van Dalen et al. (2009, 2010) either let younger and older workers be rated in comparison, or calculated difference scores between those groups. Loretto et al. (2000) and McGregor and Gray (2002), however, did not ask for comparison. There is also positive (Hassell and Perrewe, 1995; McGregor and Gray, 2002) and negative support (Loretto et al., 2000; Gringart et al., 2005; Van Dalen et al., 2009, 2010) for the willingness to train, with no apparent explanation with regard to sample, or measurement.

There are also specific stereotypes toward younger workers. Younger workers are typically perceived as positive concerning the abilities and characteristics in which older workers are seen as negative, and vice versa (Van Dalen et al., 2010). This notion refers to, for example, younger workers being seen positively regarding their technical skills (Weeks et al., 2017), flexibility, physical strength, productivity, interest in trainings, creativity (Van Dalen et al., 2009), adaptability (DeArmond et al., 2006), and learning capability (Van Dalen et al., 2010). Examples of negative stereotypes toward younger workers include characteristics such as inexperienced, unmotivated, unreliable, or arrogant (Finkelstein et al., 2012).

The specific stereotypes abovementioned can be classified into dimensions of existing conceptual frameworks. The stereotype content model (Fiske et al., 2002) holds competence and warmth as the two primary dimensions that groups are judged on (e.g., older people are seen as warm, but less competent). Marcus et al. (2016) addressed the perception of workers by adding adaptability as a third essential dimension on their work-related age-based stereotypes (WAS) scale, whereas Henkens (2005) extracted productivity, reliability, and adaptability as relevant dimensions. Altogether, performance (including competence), adaptability, reliability, and warmth are important dimensions of perceptions of the workforce that all were included in the measurement of age-related stereotypes in a recent (Kleissner and Jahn, 2020) and the present study.

Explicit age stereotypes in the workplace may be dependent on various moderating variables, such as the age of raters (Hassell and Perrewe, 1995), contact frequency (Henkens, 2005), contact quality (Iweins et al., 2013), self-categorization as a member of a certain age group (Bal et al., 2015), perception of younger or older people in general and the perceived malleability of aging (Kleissner and Jahn, 2020). Two of these moderators are of particular importance for the present research. First, against the background of social identity theory (Tajfel and Turner, 1979), which predicts a preference for the in-group because people seek a positive social identity, the age of raters is supposed to be connected with in-group favoritism. Support comes from Kleissner and Jahn (2020) who observed a more positive evaluation of one’s own group and Zaniboni et al. (2019) where explicit age stereotypes were negatively correlated with participant age. Second, the perception of the aging process as malleable is a recently found potential predictor of more positive explicit age stereotypes about older nurses (Kleissner and Jahn, 2020) and we are interested if perceived malleability of the aging process probably also reduces implicitly measured prejudice.

### Implicitly Measured Age Stereotypes in the Workplace

The examination of implicit age stereotypes in the workplace is a relatively new research area in social and organizational psychology. Whereas the explicit measurement of age stereotypes typically deals with stereotype content or moderating variables, the implicit measurement of age stereotypes is typically applied to identify the influence of implicit age stereotypes on evaluations. Zaniboni et al. (2019) measured implicit age stereotypes toward older workers with an IAT and found that negative implicit age stereotypes were connected with worse evaluations of older job applicants. Thus, implicit age stereotypes can lead to discriminatory behavior in the hiring process. Leaving the hiring context, Malinen and Johnston (2013) were the only ones who employed the IAT for investigating implicit evaluations of younger compared to older workers. The IAT revealed a more positive attitude of two samples of university students toward younger than older workers although no such attitude difference was apparent in averaged semantic differential scores as an explicit measure. However, there were two main limitations in their study. First, the context of the workplace was not that salient because only the target category was adapted (younger and older workers instead of young and old). Second, positive and negative words were derived from word lists from Nosek and Banaji (2001) and included characteristics (e.g., glad, loving) and nouns (e.g., paradise, hate) not particularly job-relevant. It could be that participants did not pay as much attention to the work-context and may have reduced “younger workers” and “older workers” to young versus old in their mind. For these two reasons, it is possible that Malinen and Johnston (2013) did not create a work-specific age IAT, but a typical age IAT with a tighter age range. Given that there is only little research on implicit measurements of age stereotypes in the workplace, we aimed at increasing the work-relatedness of an IAT to assess implicit work-related evaluations of younger and older workers. We further wanted to meet the limitation of the usual study samples by testing age-diverse samples and the target group of workers. In the next section, we briefly introduce the standard Implicit Association Test (Greenwald et al., 1998).

### The Implicit Association Test

The IAT indicates the difference in attitudes toward two target concepts (De Houwer, 2001), for instance, between the attitudes toward younger and older workers. It is an implicit measure because the difference in attitudes is inferred from response times in a categorization task that never asks for evaluating the target concepts. Participants categorize stimuli as belonging to one or the other target concept with two response keys. With the same response keys, they categorize another set of stimuli as belonging to one or the other side of an attribute dimension, for instance, positive and negative. Although stimuli from the target concepts are never categorized on the attribute dimension, the double assignment of response keys creates instances of assignments that match or mismatch with the memory association of a target concept and one side of the attribute dimension (congruent and incongruent conditions). For instance, if young workers are strongly associated with “positive” in memory and the same response key is used for “young worker” and “positive” (congruent), faster response times are expected than if the response key for “young worker” is the same as for “negative” (incongruent). The reaction time difference between congruent (e.g., young worker and positive, old worker and negative) and incongruent (e.g., young worker and negative, old worker and positive) conditions measures the relative strength of associations between target concepts and the opposite sides of the attribute dimension. If the categorization of stimuli is faster for congruent than for incongruent conditions, it is assumed that the subject prefers the associations of the congruent condition (e.g., young worker and positive, old worker and negative). As a measure of automatically activated associations that does not depend on self-report and is relatively outside the conscious control (Nosek et al., 2002), the IAT is used in most areas of psychology. For example, it is used to measure implicit consumer attitudes in market research (e.g., juices versus sodas; Maison et al., 2001), implicit attitudes in health psychology (e.g., discriminating snake and spider fear groups; Teachman et al., 2001), the development of implicit attitudes in developmental psychology (e.g., via race evaluations of different age groups; Baron and Banaji, 2006), the personality self-concept in personality psychology (e.g., targeting shyness; Asendorpf et al., 2002), or implicit attitudes toward age groups in Geropsychology (e.g., Hummert et al., 2002).

The procedure of an IAT is usually comprised of seven blocks of trials (see Table 1). In the first block, participants practice an initial response key assignment with stimuli of the target concepts (e.g., young and old workers) followed by the stimuli of the attribute dimension in the second block (e.g., positive and negative). The third and the fourth blocks are comprised of the primary combination of target and attribute items. As the response key assignment for the target stimuli is changed for the second combination, participants practice the reversed assignment with target items in block five. Blocks six and seven consist of the second combination of target and attribute items. Initially (Greenwald et al., 1998), block three and block six were intended solely as practice blocks, but in the revised scoring algorithm (Greenwald et al., 2003), all trials of blocks three, four, six, and seven are included in calculating the *D* measure. Due to similarities in calculation to Cohen (1977), the IAT measure is presented as an italicized uppercase letter (*D*). The difference is that the standard deviation is computed from the scores in both conditions instead of a pooled standard deviation (see Table 2).

**TABLE 1:** Sequence of trial blocks in the worker IAT of this study.

Block	No. of trials	Function	Items assigned to left-key response	Items assigned to right-key response
1	24	Practice	Younger workers	Older workers
2	24	Practice	Positive job characteristics	Negative job characteristics
3	24	Test	Younger workers + positive	Older workers + negative
4	48	Test	job characteristics	job characteristics
5	24	Practice	Older workers	Younger workers
6	24	Test	Older workers + positive job characteristics	Younger workers + negative job characteristics
7	48	Test		

**TABLE 2:** Scoring algorithm for *D*_2_ as presented by Greenwald et al. (2003).

1	Include all trials of Block 3, 4, 6, and 7
2	Delete trials < 400 ms
3	Delete trials > 10,000 ms
4	Exclude subjects with 10% latencies below 300 ms
5	Include error latencies
6	Compute the pooled standard deviation of Blocks 3 and 6 as well as 4 and 7
7	Compute mean latencies of each Block (3, 4, 6, 7)
8	Compute two mean difference scores (Mean_Block6_ – Mean_Block3_ and Mean_Block7_ – Mean_Block4_)
9	Divide those scores through their pooled standard deviation
10	Average both ratios

The target and attribute stimuli are selected to represent the respective concept. Semantic associations between target and attribute stimuli are usually avoided because they can strongly influence IAT results. For instance, Bluemke and Friese (2006) varied attribute and target stimuli aimed at exploring implicit attitudes about Germans from the East (former GDR) and the West of Germany so that cross-category associations arose. The variation of the target stimuli concerned the valence (positive, negative, or neutral). For example, “Baltic Sea,” “communism,” and “capitalism” were positive, negative, and neutral items for the target category of East Germans. Attribute stimuli were either positive (e.g., “hospitable” for East), or negative (e.g., “unemployed” for East). Bluemke and Friese (2006) found that minor manipulations already led to altered IAT results and thus called for careful pretesting of stimulus material. Similarly, Govan and Williams (2004) also varied the valence of the target stimuli. Using pleasant insects (e.g., butterfly) instead of unpleasant insects (e.g., flea) and unpleasant flowers (e.g., nettles) instead of pleasant flowers (e.g., rose) as target stimuli led to a reversal of the typical IAT effect (i.e., lower reaction times for the combination of flowers + pleasant attributes than for flowers + unpleasant attributes). Similarly, positive examples of Blacks (e.g., Michael Jordan) and negative examples of Whites (e.g., Hannibal Lechter) eliminated the typical superiority of Whites + pleasant attributes (Govan and Williams, 2004). Motivated by these findings, we wanted to investigate the influence of variations of the attribute stimuli. We varied the stimulus material for the subsample of workers, so that either an incongruent cross-category association existed (i.e., typical “old”-associated positive job-relevant attributes combined with young workers), or that the association of the assumed stereotype should be facilitated (i.e., typical “young”-associated positive job-relevant attributes combined with young workers). We continue with the presentation of results of age IATs and their correlation with explicit attitudes.

### Age IAT and Correlation With Explicit Attitudes

Previous research on ageism using the IAT generally shows moderate to strong preferences for young over old people (Karpinski and Hilton, 2001; Hummert et al., 2002; Nosek et al., 2002; Greenwald et al., 2003; Nosek et al., 2005; Joy-Gaba and Nosek, 2010) and moderate preferences for young over old workers (Malinen and Johnston, 2013; Zaniboni et al., 2019). However, Lin et al. (2011) found neutral implicitly measured attitudes toward older people in their sample of psychology students and reasoned with the potential greater amount of time that Australian students spend with their grandparents. Studies using the conventional scoring algorithm (Greenwald et al., 1998) mainly reported low and non-significant implicit-explicit correlations of age attitudes (all *r* = −0.06 to 0.17; Karpinski and Hilton, 2001; Hummert et al., 2002; Nosek et al., 2002), whereas studies that applied the revised scoring algorithm (Greenwald et al., 2003) reported moderate correlations (*r* = 0.14–0.26; Nosek, 2005; Joy-Gaba and Nosek, 2010). The magnitude of implicit-explicit correlations appears dependent on the similarity of the implicit and explicit measurement of attitudes (Nosek et al., 2005). The implicit-explicit correlation was highest when both implicit and explicit measures were relative measures.

### The Present Study

Both explicit and implicit measures of age stereotypes were applied to investigate attitudes underlying workplace ageism more comprehensively. We extended typical explicit measures (thermometer ratings, comparative preference rating) by a recent multidimensional work-related stereotype scale and constructed a work-related age IAT as implicit measure. We addressed three questions that are followed by the respective hypotheses:

• Are implicitly measured attitudes toward young and old workers actually shared among age-diverse samples (students and older adults) and the target group (workers)? In line with social identity theory (Tajfel and Turner, 1979) and supporting evidence from research on explicit age stereotypes (e.g., Zaniboni et al., 2019; Kleissner and Jahn, 2020) we expected a greater implicit preference for young over old workers for younger age groups (i.e., students and younger workers) and a smaller implicit age bias for older age groups (i.e., older adults, older workers).
• How are explicit and implicit measures of age stereotypes in the workplace related? In accordance with previous research (Nosek, 2005; Joy-Gaba and Nosek, 2010), we expected moderate relationships between explicit and implicit measures. We extended typical explicit measures (thermometer ratings, stereotype scale) by examining how malleable participants perceive the aging process and expected a smaller implicit age bias for participants who perceive the aging process as malleable.
• How stable is the implicit age bias toward older workers? To test the dominance and stability of the implicit age bias we varied the stimulus material. We manipulated positive, job-relevant attributes for the sample of workers so that they were either age-neutral (e.g., “communicative”), typically “older” (e.g., “experienced”) or typically “younger” (e.g., “innovative”). Hereby, we wanted to determine whether it is only the valence of the attribute stimuli that takes effect, or if participants process the whole content, that is valence and meaning, with semantic effects on IAT results. Following Bluemke and Friese (2006), we expected a stronger bias toward older workers for the condition with typical younger attributes, followed by the condition with age-neutral attributes and we anticipated the weakest bias for the condition with typical older attributes.

For clarity and because of additional conditions and material employed within the sample of workers, we report the results obtained with students (Study 1A), older adults (1B), and workers (1C) separately, and then compare these groups in an overall analysis.

## Study 1A – Students

### Method

#### Participants

Fifty psychology students (42 female) participated in the study and either received course credit or five Euros. The age of participants ranged from 18 to 39 years (*M* = 23.20, *SD* = 4.48). A power analysis of the one sample *t*-test to examine the assumed age bias with a known high effect size (*d*) of around 1.00 for age IATs (e.g., Karpinski and Hilton, 2001; Nosek et al., 2002; Malinen and Johnston, 2013) yielded a minimum sample size of thirteen (α = 0.05, 1-β = 0.95, one-sided).

#### Measures

##### Implicit Association Test

Attitudes toward older and younger workers were measured by an Implicit Association Test (IAT, Greenwald et al., 1998) using E-Prime 3.0 software (Psychology Software Tools, 2016, Pittsburgh, PA, United States). Greenwald et al. (2003) presented and discussed six alternative *D* measures. The *D*_2_ measure (see Table 2) was chosen for the current study because participants had to correct wrong responses (a red cross in the middle of the display indicated a wrong answer).

In the current study, we used twelve images of younger (18–35 years) and older (50–65 years) workers as stimuli for the target concept (three men and three women each). Because the image database search was not successful, new images were created and selected in two steps in a pretesting procedure. In the first round, 44 psychology students from three different courses (*M* = 23.66 years, *SD* = 5.27, 84.1% female) rated twelve images on a five-point Likert scale ranging from 20 years to 65 years old. Because two images were not properly assigned, we added five additional images. In the second round, 25 participants (*M* = 30.04 years, *SD* = 13.14; 72% female) were recruited on the internet and were asked to indicate how old they believed the people in the pictures were. The mean ages of the individuals portrayed in the images sat within the proposed age ranges (18–35 and 50–65). To reduce the salience of other characteristics than age, images were presented in various shades of gray and showed the face from the mouth to the eyes with neutral facial expressions.

Six positive and six negative job-relevant characteristics were used for the attribute dimension. In a pilot study, 94 positive characteristics drawn from previous studies and job advertisements were rated on age neutrality, valence, and job relevance by 50 participants each (*M* = 27.15 years, *SD* = 9.11, 80.7% female). An additional 100 participants (*M* = 31.89 years, *SD* = 13.15, 82% female) rated 27 negative characteristics on age neutrality and valence. The participants were recruited on the internet and did not receive compensation. We screened for age-neutral, positive, and negative characteristics that are job-relevant in their opposite (positive) meaning. Attributes were designated as age-neutral if less than a quarter of participants assigned them to either “people in their twenties” or “people in their sixties” and more than half assigned them to “both.” Regarding valence, attributes were rated on a five-point Likert scale ranging from very negative to very positive. We filtered *team-minded*, *confident*, *dedicated*, *collaborative*, *communicative*, and *helpful* as age neutral, positive words and *rude*, *inefficient*, *indifferent*, *uninterested*, *uncooperative*, and *underachieving* as age neutral, negative words^1^.

##### Questionnaire

After the IAT, participants completed a questionnaire on explicit work-related age stereotypes. The questionnaire contained a list of 20 job-relevant attributes that had to be rated on a five-point scale separately for younger (35 years and younger) and older (50 years and older) workers (see Kleissner and Jahn, 2020, for an extended description). High mean scores indicate positive perception. Performance (e.g., productive, motivated), adaptability (e.g., flexible, willing to change), reliability (e.g., loyal, dependable), and warmth (e.g., friendly, cooperative) were the core dimensions and consisted of five attributes each.

Malleability of aging was measured by six items (e.g., “I can influence my aging”) on a five-point scale (Cohen’s α = 0.50) similar to Kleissner and Jahn (2020) and based upon the Essentialist-Beliefs-About-Aging Scale (Weiss, 2018). The higher the mean score, the more malleable respondents perceive the aging process. To allow for comparison with measures most frequently used in age IATs (i.e., comparative preference of young and old people and separate thermometer scales for older and younger people for the attribute dimension; e.g., Greenwald et al., 2003), participants were further asked to rate how cold or warm they perceive younger and older workers (on a 11-point Likert scale from cold to warm) and to indicate which statement out of five best describes themselves regarding preferences toward younger or older workers (ordered from a strong preference for older to a strong preference for younger workers, 1–5). Participants were also asked to indicate whether they view themselves as belonging to the old (1), middle (2), or younger (3) age group. Group identification was assessed because of its association with reduced age stereotypes when participants identify with older workers (e.g., Posthuma and Campion, 2009). To measure preferences by imaginary choice, approaching measuring a behavioral component, we developed a short scenario about a hiring decision. Participants were asked to imagine that they are responsible for human resources and must coordinate a new project, “health promotion in the workplace.” They must choose between one of two colleagues who should work with them on the project. The candidates have equal qualifications but differ in age (26 and 56 years). “Uncertain” was a possible answer, so participants were not forced to choose (1 = old, 2 = uncertain, 3 = young). The questionnaire concluded with demographics.

#### Procedure

After reading the information sheet stating that the study goal was to better understand how young and old workers are perceived, participants signed an informed consent and a data protection declaration. They were randomly assigned to one of two conditions which varied in the order of IAT blocks (congruent or incongruent blocks first). Before the IAT started, participants were first introduced to the word stimuli (positive and negative job-relevant characteristics) and then familiarized with the pictures representing younger and older workers. For standardization and to avoid distraction, the experiment took place in a laboratory. Participants worked on the IAT first and completed the questionnaire afterward. To test for reliability of the IAT, participants returned for a second session after an interval of about 1 week. At the second testing date, they only retook the IAT.

### Results

#### Implicit Association Test

**FIGURE 1:**
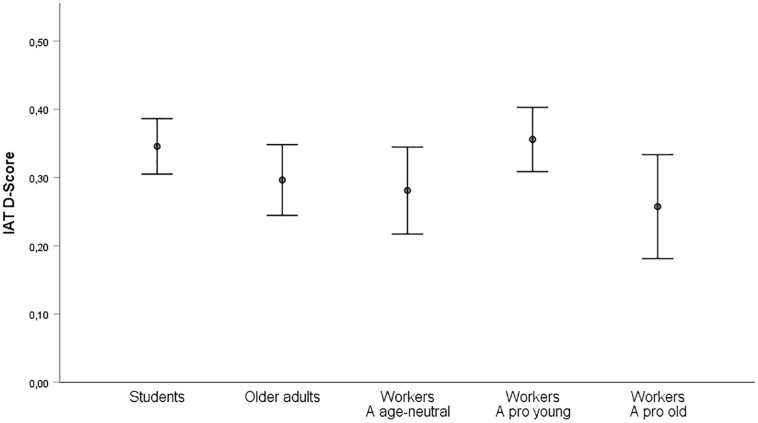
Mean *D* scores and standard errors for groups (students, older adults, workers) and experimental conditions for workers (age-neutral attribute stimuli, young-associated attribute stimuli, old-associated attribute stimuli).

Positive *D* scores indicate stronger associations of younger workers and positive (older workers and negative) relative to younger workers and negative (older workers and positive). Negative *D* scores indicate stronger associations of younger workers and negative (older workers and positive) relative to younger workers and positive (older workers and negative). The error rate (0.07) was comparable with previous studies (e.g., Karpinski and Hilton, 2001; Malinen and Johnston, 2013). With mean *D* scores of 0.34 (*SD* = 0.29) and 0.35 (*SD* = 0.35) for test and retest, respectively, the IAT revealed more positive attitudes toward younger than older workers in both sessions with *t*(49) = 8.28, *p* < 0.001, *d* = 1.17, and *t*(49) = 6.98, *p* < 0.001, *d* = 0.99, respectively (Table 3 and Figure 1). Order of block presentation was relevant. Participants who worked on congruent trials first had significantly higher *D* scores (0.43) than participants who worked on incongruent trials first (0.26, see Table 3). The test–retest reliability was 0.57 for participants with a test-retest interval of exactly 1 week (*n* = 37), and 0.50 for all participants (*M* = 8.28 days, *SD* = 3.36, 4 days minimum, 21 days maximum). The split-half reliability was calculated by correlating the difference scores of Block6/3 and Block7/4 (see Table 2). We applied a Spearman-Brown correction and found a split-half reliability of 0.89.

**TABLE 3:** Means, standard deviations, and *t*-tests of *D* measure of students, older adults and workers.

Sample	IAT	*n*	Congruent–Incongruent		Incongruent–Congruent		*t(df)*	*p*	*d*
			*M*	*SD*	*M*	*SD*			
Students	Age-neutral	50	0.43	0.30	0.26	0.27	2.13(48)	0.039	0.58
	Age-neutral Retest	50	0.54	0.24	0.15	0.34	4.72(48)	< 0.001	1.12
Older adults	Age-neutral	53	0.26	0.39	0.29	0.38	−0.29(51)	0.775	0.08
Workers	Age-neutral	31	0.23	0.39	0.33	0.31	−0.80(29)	0.428	0.29
	Positive_young	31	0.38	0.32	0.34	0.20	0.40(29)	0.692	0.14
	Positive_old	31	0.30	0.50	0.21	0.33	0.59(29)	0.558	0.21

#### Explicit Measures

Younger workers were rated higher on the mean of the entire stereotype scale than older workers (both α = 0.82, *d* = 0.93) and older workers received higher warmth-ratings than younger workers (*d* = 0.78, see Table 4). Because the extremes of the five preference statements were hardly ever chosen, we report aggregated results. According to the preference statements, 36.0% moderately or strongly preferred younger workers, 24.0% moderately or strongly preferred older workers and 38.0% reported to equally like younger and older workers (2.0% missing). In the imaginary scenario, 20.0% chose the older worker, 34.0% chose the younger worker, and 44.0% were uncertain (2.0% missing). Students perceived the aging process as rather malleable (*M* = 3.70, *SD* = 0.56), and identified themselves as belonging to the young age group (94.0%).

**TABLE 4:** Means, standard deviations, and *t*-tests of explicit measures.

Sample	Variable	Younger workers		Older workers		*t(df)*	*p*	*d*
		*M*	*SD*	*M*	*SD*			
Students	Stereotypes	3.74	0.32	3.48	0.34	−6.56(49)	< 0.001	0.93
	Warmth	7.08	1.30	8.24	1.35	5.46(48)	< 0.001	0.78
Older adults	Stereotypes	3.73	0.38	3.84	0.41	1.99(52)	0.052	0.28
	Warmth	6.46	1.64	8.54	1.11	8.36(52)	< 0.001	1.16
Workers	Stereotypes	3.56	0.36	3.55	0.39	−0.41(91)	0.686	0.04
	Warmth	6.38	1.37	7.75	1.57	6.88(91)	< 0.001	0.72

#### Implicit–Explicit Correlations

We computed difference scores for ratings of younger and older workers regarding warmth and stereotypes to correlate them with the *D* measure obtained in the first session (for comparability with the remaining samples, who completed one session only). Correlations with warmth [*r*(47) = 0.10, *p* = 0.25], stereotypes [*r*(48) = 0.07, *p* = 0.31], and malleability of aging [*r*(47) = −0.19, *p* = 0.09] were small, however, the correlation between the preference-rating and the relative implicit measure was higher [*r*(47) = 0.24, *p* = 0.05]. Participants who preferred younger workers showed stronger positive implicit associations toward younger relative to older workers. The choice of worker in the scenario [*r*(47) = 0.03, *p* = 0.41], and the group identification [*r*(47) = 0.06, *p* = 0.33] were not associated with the implicit measure.

### Discussion

The results revealed moderate implicitly measured preferences for younger workers (*D* = 0.34) which is comparable to some age IATs (e.g., 0.31, Joy-Gaba and Nosek, 2010; 0.36–0.41, Malinen and Johnston, 2013) but lower than in others (e.g., 1.25 and 1.39 for *D*_2_, Greenwald et al., 2003; 0.56, Nosek, 2005; 0.44–0.65, Nosek et al., 2005). Presumably, this is due to smaller age differences between older and younger workers in the current IAT than in typical age IATs that assess the evaluations of younger and older people. Order of blocks mattered. Participants who dealt with congruent tasks first had a stronger bias than participants who dealt with incongruent tasks first. This phenomenon was observed many times and led to the recommendation of counterbalancing the block order (e.g., Nosek et al., 2005). The split-half reliability is satisfactory (cf. Greenwald and Nosek, 2001; De Houwer and De Bruycker, 2007; Gattol et al., 2011), however, the test–retest reliability is not satisfactory with 0.57, but consistent with previous findings (e.g., Cunningham et al., 2001; Malinen and Johnston, 2013), and likely still better than for other implicit measures (Bosson et al., 2000). Unexpectedly, almost all explicit measures were uncorrelated with the implicit measure. The only significant correlation was observed for preference rating, as the more participants explicitly preferred younger workers, the more they preferred them according to the IAT.

In sum, students preferred younger workers over older workers according to the implicit measure. The explicitly measured attitudes were partly positive toward older workers, who were judged higher on warmth but lower on the overall mean of the stereotype scale. We were interested, whether the results would change if we looked at the other extreme: older adults.

## Study 1B – Older Adults

### Method

#### Participants

Fifty-five older adults (33 female, *M* = 70.42 years, *SD* = 6.27, 59 to 90 years) participated in the study and received five Euros for compensation. Due to large error rates (0.23 and 0.31) and difficulty in operating the computer, two participants were excluded from further analyses. Older adults were recruited through newspaper advertisement and local notice boards.

#### Measures and Procedure

Measures (IAT and questionnaire) and procedure were similar to Experiment 1, but there was just one test session.

### Results

#### Implicit Association Test

The error rate was low (0.04). With a mean score of 0.28 (*SD* = 0.38) for the entire sample, *D* differed significantly from zero [*t*(52) = 5.35, *p* < 0.001, *d* = 0.73; see Table 3]. The order of blocks made no difference regarding the *D* measure (see Table 3). The split-half reliability was 0.82.

#### Explicit Measures

Older workers (α = 0.87) were rated slightly higher on the mean of the stereotype scale than younger workers (α = 0.84, *d* = 0.28) and clearly higher on warmth (*d* = 1.16, see Table 4). Regarding the preference rating, 15.1% moderately or strongly preferred younger workers, 9.5% moderately or strongly preferred older workers, and 71.7% reported to have no preference (3.8% missing); 49.1% chose the younger worker in the scenario, 39.6% chose the older worker, and 11.3% were uncertain. Most older adults saw themselves as belonging to the old age group (73.6%), and perceived the aging process as rather malleable (*M* = 3.63, *SD* = 0.58).

#### Implicit-Explicit Correlations

The *D* score hardly correlated with the difference score of stereotype ratings [*r*(50) = 0.18, *p* = 0.11]. Malleability of aging was related to smaller *D* values [*r*(51) = −0.26, *p* = 0.03], and, surprisingly, subjects who preferred older workers explicitly and those who rated older workers warmer showed a higher implicitly measured preference for younger workers [*r*(49) = −0.32, *p* = 0.01, and *r*(50) = −0.43, *p* = 0.001, respectively]. There was no connection between the implicit measure and the choice of worker in the scenario [*r*(51) = −0.02, *p* = 0.43], and only a small decrease of bias for the self-assignment to the old age group [*r*(51) = −0.14, *p* = 0.17].

### Discussion

Similar to previous age IATs (e.g., Nosek et al., 2002, 2007), older adults preferred younger workers over older workers according to the IAT (*D* = 0.28). They rated older workers slightly better on the overall mean of the stereotype scale than younger workers, which might be due to an in-group bias if the shared social category of age was dominant (Turner et al., 1979). Approximately two-thirds reported no preference for any group. The implicitly measured preference for younger workers was lower the more positively older adults perceived the aging process. Surprisingly, the implicitly measured preference for younger workers was moderately associated with an explicit preference for older workers as well as with higher warmth ratings of older workers. These correlations are reversed compared to the ones observed in the student sample. It is possible that those with higher implicitly measurable bias toward older workers noticed their preference through self-observation and, therefore, wanted to undo any bias when explicitly asked. Neither explicit nor implicit measures were associated with the choice of worker in the scenario.

Both extreme groups with regard to age (students and older adults) showed negative implicit attitudes toward older workers. It remains to be seen how workers of varying age perceive older and younger workers according to implicit and explicit measures. Two additional IAT conditions were created to explore the stability of age biases against semantic influence from cross-category associations.

## Study 1C – Active Workers and Semantic Influences in the IAT

### Method

#### Participants

Ninety-three workers (47 female, *M* = 38.97 years, *SD* = 12.46, 17–64 years) participated in this study. They were recruited through social media and direct requests to their firms asking whether employees would be exempted for participating. Participants were randomly allocated to one of three conditions and received five Euros for compensation.

#### Measures and Procedure

##### Implicit Association Test

We wanted to determine whether the implicit preference for younger workers is also prevalent in the target group of workers. Furthermore, we are interested in how reaction times might change if the stimulus material is biased toward younger workers or older workers. In the first condition (Positive_neutral), participants worked through the same materials as students and older adults. For the second (Positive_young) and third conditions (Positive_old), positive age-neutral job-relevant words were exchanged with age-specific ones that were drawn from the same word lists as described in Study 1A. *Flexible*, *innovative*, *energetic*, *dynamic*, *willing to change*, and *curious* were filtered as positive job-relevant attributes associated with younger workers (*M* = 1.12–1.44), and *proficient*, *experienced*, *patient*, *prudent*, *reliable*, and *conscientious* as positive and associated with older workers (*M* = 2.46–3.00). Regarding age-specific words for younger workers, all attributes were more clearly ascribed to younger than older workers and had a mean score between 1.12 and 1.44 (1 = younger workers, 2 = both, 3 = older workers). Mean scores for attributes associated most with older workers sat between 2.46 and 3.00. The negative attributes in all three conditions remained the same age-neutral words as in Studies 1A and 1B. The sessions were conducted in quiet environments (e.g., an office).

##### Questionnaire

Workers received the same questionnaire as students and older adults. They were additionally asked to indicate how often they have contact with younger and older workers (1 = occasionally, 5 = daily) and if they regard themselves as older (1), middle-aged (2), or younger workers (3). The procedure was identical to Study 1B.

### Results

#### Implicit Association Test

The error rate (0.05) sat within the normal range. *D* scores (see Table 3 and Figure 1) significantly differed from zero for the Positive_neutral condition, *t*(30) = 4.41, *p* < 0.001, *d* = 0.79, the Positive_young condition, *t*(30) = 7.56, *p* < 0.001, *d* = 1.36, and the Positive_old condition, *t*(30) = 3.38, *p* = 0.002, *d* = 0.61, but there were no order effects (see Table 3). *D* was highest for Positive_young (*M* = 0.36, *SD* = 0.26), followed by Positive_neutral (*M* = 0.28, *SD* = 0.35), and Positive_old (*M* = 0.26, *SD* = 0.42). Because of heterogeneous variances, a Kruskal–Wallis test was performed. The differences between worker groups were non-significant [*H*(2) = 1.68, *p* = 0.43]. To test the prediction of in-group favoritism, workers were categorized in three age groups: younger workers (35 years and younger), middle-aged workers (36–49 years), and older workers (50 years and older). The *D* score did not vary as a function of age group for the Positive_neutral condition [*F*(2,28) = 0.77, *p* = 0.47, η^2^ = 0.05], the Positive_young condition [*F*(2,28) = 0.61, *p* = 0.55, η^2^ = 0.04], and the Positive_old condition [*F*(2,28) = 1.19, *p* = 0.32, η^2^ = 0.08]. The split-half reliability was 0.54 for the Positive_neutral condition, 0.89 for the Positive_young condition, and 0.86 for the Positive_old condition.

#### Explicit Measures

Because explicit measures did not vary across conditions, we report them for the entire sample of workers. There was no difference regarding stereotype ratings of younger and older workers (both α = 0.83; *d* = 0.04), but older workers were rated higher on warmth (*d* = 0.72, see Table 4). Regarding explicit age preference, 18.3% reported to moderately or strongly prefer younger workers, whereas 16.1% preferred older workers, and 64.5% reported not having any preference (1.1% missing). With regard to the scenario, 38.7% chose the younger worker, 37.6% chose the older worker, and 21.5% reported to be uncertain (2.2% missing). In the self-assignment to age groups, 35.5% identified themselves as younger workers, 52.7% as middle-aged workers, and 8.6% as older workers (3.2% missing). The general age group assignment revealed very similar percentages [*r*(88) = 0.93, *p* > 0.001]. Most participants reported daily contact with older workers (74.2%) as well as with younger workers (80.6%), and saw the aging process as rather malleable (*M* = 3.66, *SD* = 0.51).

#### Implicit–Explicit Correlations

For the implicit–explicit correlations we report results on the basis of the age-neutral IAT (for comparability with the other samples). The implicit measure did not reliably correlate with the stereotype scale, the warmth rating, the preference rating, the scenario choice, the malleability of aging, and the working or general group identification (all *p*-values > 0.09)^2^. Because variances of contact frequencies were small, correlations with the implicit measure were not calculated.

### Discussion

We observed slight to medium implicitly measured preferences for younger workers (*D* = 0.26–0.36). Unexpectedly, the implicitly measured bias did not reliably vary in strength between conditions, which means that cross-category associations in positive attributes that could have favored either young or older workers in the IAT did not exert influence as opposed to Bluemke and Friese (2006). The implicitly measured preference for younger workers was stable even if the stimulus material was biased toward older workers in the Positive_old condition. Thus, either participants only regarded the valence of attribute stimuli as instructed and did not process the word content much further, or the bias from word content in Positive_young and Positive_old conditions was not strong enough. Inconsistent with the prediction of in-group bias derived from social identity theory (Tajfel and Turner, 1979), the implicit bias did not differ for age groups. The implicit-explicit correlations were not reliable. The explicit findings were almost bias-free, with the exception that older workers were perceived more warmly than younger workers. Due to small variances, we were not able to examine the contact hypothesis (Allport, 1954) that predicts lower biases toward a group with increased contact with its members.

In the next section we will examine whether the samples (students, older adults, workers) differ and we will further analyze the stereotype scale.

## Overall Calculation

Means, standard deviations, and correlations for the study variables on the basis of age-neutral IATs are presented in Table 5.

**TABLE 5:** Means, standard deviations, and correlations of implicit and explicit measures.

Variable	*M*	*SD*	(1)	(2)	(3)	(4)	(5)	(6)
(1) *D* score^a^	0.30	0.34						
(2) Stereotype scale^b^	0.06	0.39	0.13					
(3) Warmth-rating^b^	–1.67	1.71	–0.12	0.21**				
(4) Preference^c^	3.08	0.79	0.02	0.18*	0.22**			
(5) Group identification^d^	2.15	0.87	0.06	0.35**	0.31**	0.14		
(6) Malleability of aging^e^	3.66	0.54	−0.20*	–0.13	0.09	–0.09	0.07	
(7) Scenario^f^	1.98	0.86	0.01	0.23**	0.08	0.19*	0.14	−0.06

Only results from the age-neutral IATs were included (n = 131–134). ^a^Positive values indicate stronger association between younger workers and positive (older workers and negative) compared to younger workers and negative (older workers and positive). ^b^Positive values mean that younger workers are rated more positively than older workers, negative values mean that older workers are rated more positively than younger workers. ^c^1 = strong preference for old workers; 5 = strong preference for young workers. ^d^1 = old; 2 = middle-aged; 3 = young. ^e^1 = immutable; 5 = malleable. ^f^1 = old worker; 2 = uncertain; 3 = young worker. *p < 0.05, **p < 0.01.

### Implicit Association Test

The overall error rate was 0.05. To test the influence of the current occupation (students, workers, older adults), we calculated an ANOVA (assumptions have been met) on the basis of the age-neutral IATs. The *D* score did not vary as a function of sample [*F*(2,114) = 0.03, *p* = 0.97, $\eta_{\text{p}}^{2}$ = 0.001; see Figure 1], order [*F*(1,114) = 0.30, *p* = 0.58, $\eta_{\text{p}}^{2}$ = 0.003], gender [*F*(1,114) = 0.50, *p* = 0.48, $\eta_{\text{p}}^{2}$ = 0.004], or age group [*F*(2,114) = 0.09, *p* = 0.91, $\eta_{\text{p}}^{2}$ = 0.002]. There were no significant interactions but sample × gender [*F*(2,114) = 4.36, *p* = 0.02, $\eta_{\text{p}}^{2}$ = 0.07] with higher *D* scores for women than men in the sample of older adults). The pooled split-half reliability was 0.83.

### Explicit Measures

Older and younger workers were rated similarly positive on the stereotype scale (Cohen’s α = 0.85 and 0.84, respectively), *t*(193) = −1.70, *p* = 0.09, *d* = 0.12, and older workers were rated much warmer than younger workers, *t*(192) = 11.60, *p* < 0.001, *d* = 0.83, but there were significant differences between participant groups [stereotype scale difference scores: *F*(2,191) = 12.90, *p* < 0.001, η^2^ = 0.12, and warmth rating difference scores: *F*(2,190) = 3.85, *p* = 0.02, η^2^ = 0.04, respectively]. *Post hoc* comparisons using the Tukey HSD demonstrated that students rated younger workers more positively than older workers (*M* = 0.26, *SD* = 0.28), whereas older adults rated older workers more positively than younger workers (*M* = −0.10, *SD* = 0.37). Workers rated older and younger workers equally positively (*M* = 0.02, *SD* = 0.41). With regard to warmth, students also favored older workers (*M* = −1.16, *SD* = 1.49), but not as strongly as older adults favored older workers (*M* = −2.08, *SD* = 1.79). A single sample *t*-test against 3 (neutral rating) revealed no preference for younger or older workers, *t*(191) = 1.23, *p* = 0.22, *d* = 0.09, and a Kruskal–Wallis test confirmed that this is true for all three groups [*H*(2) = 1.06, *p* = 0.59]. In the imaginary scenario, 36.7% chose the older worker and 37.2% chose the younger worker (24.5% reported to be uncertain, 1.5% were missing); a Kruskal–Wallis test showed no effect of participant group, *H*(2) = 1.93, *p* = 0.38. Moreover, the samples did not differ in regard to their perception of the malleability of aging [*M* = 3.66, *SD* = 0.54; *F*(2,191) = 0.19, *p* = 0.83, η^2^ = 0.002].

**FIGURE 2:**
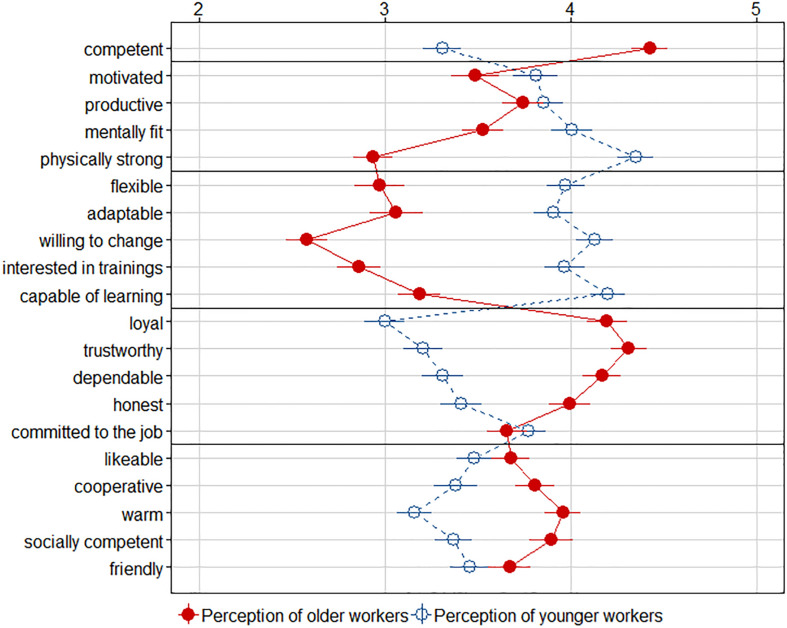
Means and 95% confidence intervals for work-related age stereotypes of younger and older workers. Horizontal lines separate the dimensions of performance, adaptability, reliability, and warmth. The item “competent” was analyzed separately from the performance dimension. We found stable explicit attitudes: Younger workers (dashed lines) were rated higher on the dimensions of performance andadaptability, whereas older workers (solid lines) were rated higher on competence and on the dimensions of reliability and warmth.

Even though older and younger workers were rated similarly on the overall mean of the stereotype scale, we observed interesting findings on the level of individual dimensions (see Figure 2). Regarding attributes of the performance dimension, for all but the attribute “competent,” higher ratings were obtained for younger workers (as in Kleissner and Jahn, 2020). For “competent,” older workers received higher ratings. Thus, the mean performance rating was computed without the competent-rating that is reported separately. We calculated a confirmatory factor analysis using Maximum Likelihood estimation through MPlus 8 to check the grouping of attributes in dimensions. Similar to Kleissner and Jahn (2020), the fit indices [χ^2^(164) = 386.21, *p* < 0.001, CFI = 0.81, RMSEA = 0.08 [0.07,0.09], SRMR = 0.08] were marginally acceptable. Younger workers were rated higher on performance (*d* = 0.72) and adaptability (*d* = 1.30), whereas older workers were rated higher on reliability (*d* = 1.32), warmth (*d* = 0.80), and the single characteristic “competent” (*d* = 1.14) (see Figure 1). All three samples (students, older adults, workers) showed a similar pattern, although there were slight differences in the effect sizes for performance [*F*(2,191) = 16.45, *p* < 0.001, η^2^ = 0.15], adaptability [*F*(2,191) = 4.62, *p* < 0.011, η^2^ = 0.05], and warmth [*F*(2,191) = 3.99, *p* < 0.020, η^2^ = 0.04]. *Post hoc* comparisons using the Tukey HSD indicated that students rated younger workers in relation to older workers much better on performance than older adults and workers, and higher on adaptability and warmth than older adults.

### Implicit–Explicit Correlations

The perception of the aging process as malleable was connected with a slighter implicitly measured bias [*r*(131) = −0.20, *p* = 0.01; see Table 5]. All other explicit variables did not reliably correlate with the implicit bias: stereotype scale [*r*(131) = 0.13, *p* = 0.07], warmth rating [*r*(131) = −0.12, *p* = 0.08], preference rating [*r*(131) = 0.02, *p* = 0.39], scenario choice [*r*(131) = 0.01, *p* = 0.47], and group identification [*r*(131) = 0.06, *p* = 0.24].

### Discussion

Students, older adults, and workers preferred younger over older workers according to IAT results, which is in accordance with other age IATs (e.g., Nosek et al., 2007). The strength of the implicitly measured bias did not reliably vary as a function of sample, gender, age, or block order. The results of the stereotype scales revealed slight differences between samples in the sense of in-group favoritism (Turner et al., 1979), but the detailed profile of the explicit stereotypes remained stable (i.e., older workers were seen as competent, reliable and warm, and younger workers were seen as performance-capable and adaptable). Students rated younger workers higher on the stereotype scale than older workers, whereas older adults rated older workers higher than younger workers. The group of workers rated younger and older workers equally high on the stereotype scale. All participants rated older workers warmer than younger workers. In all three groups, we found no clear explicit preference for one age group over another. There was only one significant implicit-explicit correlation: Participants who regarded the aging process as malleable showed lower implicitly measured age biases.

## General Discussion

In this study, we set out to extend the empirical basis about age attitudes and age stereotypes in the context of work and to improve their implicit and explicit measurement. With an Implicit Association Test that used pictures of young and old workers as target stimuli and positive and negative job-related characteristics as attribute stimuli, we intended to establish the context of work for implicitly measuring attitudes about young relative to old workers. A slight to moderate implicitly measured preference (*D* = 0.26–0.38) for younger workers was found in three samples: students, workers, and retired older adults. The *D* scores are comparable to some age IATs (e.g., 0.31, Joy-Gaba and Nosek, 2010; 0.36–0.41, Malinen and Johnston, 2013) but lower than in others (e.g., 1.25 and 1.39 for *D*_2_, Greenwald et al., 2003; 0.56, Nosek, 2005; 0.44–0.65, Nosek et al., 2005). The strength of the implicitly measured bias did not reliably vary as a function of sample, gender, age, or block order, corresponding with Malinen and Johnston (2013). Lin et al. (2011) also found no effects of gender and task order. In most reported age IATs, no increase in positivity of implicitly measured age attitudes toward older people with increasing age was observed (e.g., Nosek et al., 2002). In fact, Nosek et al. (2007), who examined over 351,204 age IATs between 2000 and 2006 on the website http://implicit.harvard.edu/, showed that older adults (60 and older) favored younger adults similarly strongly than did other age groups (grouped by decade from 10 to 50). Some studies (Hummert et al., 2002; Chopik and Giasson, 2017) even found that implicitly measured preferences for young adults were highest among older adults. The stable implicit bias across different worker age groups is inconsistent with in-group bias as predicted by social identity theory (Tajfel and Turner, 1979); however, it can be explained by the stereotype embodiment theory (Levy, 2009) that suggests that age stereotypes are initially developed in early childhood through the exposition to such stereotypes by the cultural environment, which presumably is and was very similar for the study participants. The test–retest reliability is not satisfactory with 0.57, but consistent with previous findings (e.g., Cunningham et al., 2001; Malinen and Johnston, 2013), and likely still better than for other implicit measures (Bosson et al., 2000). All split-half reliabilities (*r* = 0.82–0.89) are satisfactory and comparable with previous findings (e.g., De Houwer and De Bruycker, 2007; Gattol et al., 2011), except for the split-half reliability of the Positive_neutral condition in the sample of workers (*r* = 0.54).

Unexpectedly, the manipulation of the stimulus material in the sample of workers intended to create cross-category associations between target and attribute stimuli (Bluemke and Friese, 2006) only showed a tendency toward the expected effects. This result underlines the strength and stability of the implicit age bias. The implicitly measured bias remained stable whether we used age-neutral, young-associated or old-associated positive, job-relevant attribute stimuli. It is possible that participants processed the words as far as they knew their valence, but that the content was not processed much further or the age-association was too weak. At least, we observed a trend consistent with expectations. The mean *D*-score was highest with young-associated words, followed by age-neutral and old-associated words with increasing variance. Hence, we assume that the words were properly semantically processed. Bluemke and Friese (2006) obtained strong semantic effects when they manipulated both positive and negative attributes. It was only the positive attributes that varied in the current study and negative attributes remained age-neutral. Hence, manipulating both positive and negative attributes could have produced more reliable effects of cross-category associations.

Several explicit measures of attitudes and stereotypes were employed with all three samples. Explicit measures of general age preferences showed no clear age preference and only a slight bias in favor of one’s own age group. Yet, differentiated explicit measures of work-related age stereotypes (competence, performance, adaptability, reliability, warmth) revealed clear age stereotypes in all three samples: Younger workers were rated higher on performance and adaptability, older workers were seen as more competent and were rated higher on dependability and warmth. This finding was stable across groups and is congruent with previous research examining the same stereotype scale across age groups in the nursing profession (Kleissner and Jahn, 2020). The described pattern of the stereotype scale likewise corresponds with the stereotype content model in which older people are rated as warm but less competent (in the sense of performance-capable) (Cuddy et al., 2007). In the stereotype content model, competence is understood in a broad sense, therefore corresponding to the performance dimension in the current study and not the single attribute. Students rated younger workers higher on the stereotype scale than older workers, whereas older adults rated older workers higher than younger workers, which might be due to an in-group bias if the shared social category of age was dominant (Turner et al., 1979). The group of workers rated younger and older workers equally high on the stereotype scale. The imaginary choice paradigm yielded no clear preference for either the young or the old worker in all samples. Although this is a pleasing result, we assume that the preceding age-related measures sensitized the participants putting the validity of this measure into question. Furthermore, it is questionable whether the “uncertain” choices represent equal consideration of each worker or socially desirable answers. Evidence that implicit attitudes can indeed impact real decisions if social desirability is alleviated by a temporal separation of measuring implicit biases and real decisions comes from Régner et al. (2019), who examined the influence of implicit gender biases in real hiring committees in the scientific field. Régner et al. (2019) found that committees with strong implicit gender biases promoted fewer women 1 year later when they did not believe that systematic biases exist. The authors conclude that committee members probably felt less pressure to behave socially desirable after 1 year. The implicit–explicit correlations were relatively low or non-significant and support the view that implicit and explicit measures address distinct dimensions of attitude constructs (e.g., Hummert et al., 2002; Nosek et al., 2007). We were specifically interested if the perception of the aging process as malleable influences the implicitly measurable age bias, since it was a potential predictor for stereotypes toward older nurses in a recent study (Kleissner and Jahn, 2020). The perception of the aging process as malleable was related to a smaller implicit bias for the student sample and the sample of older adults, but not for the target group of workers.

We presume that we were successful in implicitly measuring age attitudes toward workers and not just general age attitudes. There are many studies using age IATs to examine preferences for older or younger people (e.g., Nosek et al., 2002) but there are only two studies (Malinen and Johnston, 2013; Zaniboni et al., 2019) that specifically addressed younger and older workers in age IATs. Consistent with our results, Malinen and Johnston (2013) and Zaniboni et al. (2019) observed moderate stable implicitly measured preferences for younger workers. To increase the salience of the work context, we carefully selected the positive and negative attribute stimuli to be job-relevant (e.g., team-minded, collaborative). We tested not just students but older adults as age-diverse samples and tested the significant target group of workers under standardized conditions. We further varied the stimulus material for the subsample of workers, so that either an incongruent cross-category association existed (i.e., typical “old”-associated positive job-relevant attributes combined with young workers), or that the association of the assumed stereotype should be facilitated (i.e., typical “young”-associated positive job-relevant attributes combined with young workers).

The fact that we obtained lower *D*-scores than typically found in general age IATs (e.g., Greenwald et al., 2003; Nosek, 2005; Nosek et al., 2005) could suggest that we (Malinen and Johnston, 2013) have not just conducted general age IATs, however, the lower *D*-scores can be explained with the smaller age differences between the younger and older workers depicted in the target stimuli. In the present study, “older” referred to people from the age of 50–65 years, whereas in typical age IATs “older” refers to retired people with more wrinkles (see Nosek et al., 2007). Thus, we conclude that our results demonstrate influences of the strength of age cues in target stimuli in age IATs.

### Practical Implications

Whereas the explicit measurement of age stereotypes revealed a differentiated picture of age stereotypes toward young and older workers, the implicit measurement assessed a general evaluation of younger compared to older workers. In the workplace, it is important to address both forms of measurement, because (discriminatory) behavior can occur as a result of conscious and unconscious processing. For human resources and specifically for age diversity trainings, it is recommended to raise awareness for both forms of age stereotypes. It is particularly important to train managers in identifying and handling age stereotypes because of their role modeling positions (Kunze et al., 2013) and their decision-making authority. Following legal regulations (e.g., ADEA, 1967; European Communities, 2000), organizational decisions (e.g., hiring, promotion) have to be bias-free and should only be based on job-relevant information (Posthuma and Campion, 2009), which is why it is essential to raise awareness to possible unconscious age biases (e.g., Zaniboni et al., 2019). For companies, it is further indispensable to include age in their policies for laying the foundation of an age-friendly climate. In addition to raising awareness for the conscious and unconscious nature of attitudes and stereotypes, specific discussion topics for diversity trainings can be derived from both forms of measurement. How can it be explained, that people report no explicit preferences for one over the other age group, but still show an implicit preference? Why do younger and older workers differ on specific stereotype dimensions? Are the stereotypes consistent with empirical evidence? The malleability of aging could be an effective topic for age diversity trainings. With regard to our samples of students and older adults, perceiving the aging process as malleable was related to a smaller implicit bias, however, it remains to be seen whether a malleability conviction indeed reduces implicit and explicit prejudices in the workplace. With addressing topics and questions like these, people can be led to reflect on their attitudes, perhaps change discriminatory attitudes in the long run, and, most importantly, consciously decide to counteract their spontaneous behavior.

### Limitations and Future Directions

We are aware that our research has two main limitations. The first is that we cannot be sure whether the age-related differences of the explicit attitudes represent changes over the life-span, or whether they have to be attributed to cohort effects due to the cross-sectional design. The second is that the context of the workplace could have been more salient regarding the target stimuli. We used pictures of younger and older workers for displaying those categories, but there was no further work-relatedness apparent in the pictures. Using pictures instead of words to represent the target category is associated with lower age IAT effects (e.g., Nosek et al., 2002); therefore, future research could examine whether there are applicable work-relevant target word stimuli (e.g., “young professional”). Moreover, we wonder whether the differentiated explicit stereotype dimensions can also be measured implicitly. Relevant attribute dimensions can be warm–cold, adaptable–unadaptable, reliable–unreliable, and competent–incompetent. However, keeping in mind that the stimulus material needs to be free of the regarded stereotype for a reliable IAT, the difficulty lies in the stereotypicality of those dimensions.

### Conclusion

Our results highlight the spread and the nature of explicitly and implicitly measurable age stereotypes and age attitudes in the workplace. Regardless of age or group, younger workers were moderately preferred according to the implicit measure. The explicit preferences were stable across samples and age of respondents in their profile. Younger workers were preferred in regard to the dimensions of performance and adaptability, and older workers were preferred in regard to competence and the dimensions of reliability and warmth. Explicit attitudes toward older workers become more positive with age, but it is alarming that regardless of age and of (biased) stimulus material, younger workers were always implicitly preferred. Our results underline the usefulness of implicit measures when looking at age attitudes for full comprehension. It is important to detect negative implicit age attitudes since they can lead to discrimination and impaired productivity. By raising awareness, implicit and differentiated explicit measures can be a first step toward counteracting ageism.

## Data Availability Statement

The raw data supporting the conclusions of this article will be made available by the authors, without undue reservation.

## Ethics Statement

The studies involving human participants were reviewed and approved by the ethics committee of the Faculty of Behavioral and Social Sciences of Chemnitz University of Technology. The patients/participants provided their written informed consent to participate in this study.

## Author Contributions

VK and GJ contributed to the conception and design of the study, interpreted the results. VK collected the data and performed the statistical analyses under the supervision of GJ. VK wrote the first draft of the manuscript. GJ provided critical revisions. Both authors contributed to the article and approved the submitted version.

## Conflict of Interest

The authors declare that the research was conducted in the absence of any commercial or financial relationships that could be construed as a potential conflict of interest.

## Footnotes

1 Please note that the experiment was conducted in German and attributes are translated (see Supplementary Material for the original words). Chosen words were examined for consistency of word length and word beginning to reduce the influence of recoding processes (Rothermund et al., 2009).

2 For the Positive_young condition, only the preference statement and the scenario choice reliably correlated with the implicit measure [*r*(29) = 0.32, *p* = 0.04, and *r*(28) = 0.44, *p* = 0.008, respectively]. For the Positive_old condition, reliable correlations with the implicit measure were found for the stereotype scale [*r*(28) = 0.37, *p* = 0.02], the general group identification [*r*(28) = 0.39, *p* = 0.02], and the working group identification [*r*(27) = 0.38, *p* = 0.02].
