# Supplementary material for: Implicit and Explicit Measurement of Work-Related Age Attitudes and Age Stereotypes
Source: Front Psychol. 2020 Oct 6;11:579155. doi: 10.3389/fpsyg.2020.579155 (PMC7573151; doi:10.3389/fpsyg.2020.579155)
Supplement: Supplementary file 1 [file Table_1.DOCX]

Appendix A

# Table 1

# *Original and Translated Word Stimuli of the Implicit Profiles*

| Age-neutral, positive attributes | |  | Positive attributes associated with younger workers | |  | Positive attributes associated with older workers | |  | Age-neutral, negative attributes | |
| --- | --- | --- | --- | --- | --- | --- | --- | --- | --- | --- |
| German | English |  | German | English |  | German | English |  | German | English |
| Teamfähig | Team-minded |  | Flexibel | Flexible |  | Erfahren | Proficient |  | Rücksichtslos | Rude |
| Selbstbewusst | Confident |  | Innovativ | Innovative |  | Routiniert | Experienced |  | Ineffizient | Inefficient |
| Engagiert | Dedicated |  | Energiegeladen | Energetic |  | Geduldig | Patient |  | Gleichgültig | Indifferent |
| Kooperativ | Collaborative |  | Dynamisch | Dynamic |  | Umsichtig | Prudent |  | Desinteressiert | Uninterested |
| Kommunikativ | Communicative |  | Veränderungsbereit | Willing to change |  | Seriös | Reliable |  | Unkollegial | Uncooperative |
| Hilfsbereit | Helpful |  | Wissbegierig | Curious |  | Pflichtbewusst | Conscientious |  | Leistungsschwach | Underachieving |
